# A comparison of the bone and growth phenotype of *mdx*, *mdx:Cmah^−/−^* and *mdx:Utrn^+/−^* murine models with the C57BL/10 wild-type mouse

**DOI:** 10.1242/dmm.040659

**Published:** 2020-01-10

**Authors:** Claire L. Wood, Karla J. Suchacki, Rob van ’t Hof, Will P. Cawthorn, Scott Dillon, Volker Straub, Sze Choong Wong, Syed F. Ahmed, Colin Farquharson

**Affiliations:** 1 Division of Developmental Biology, Roslin Institute, University of Edinburgh, Midlothian, EH25 9RG, UK; 2 John Walton Muscular Dystrophy Research Centre, Newcastle University and Newcastle Hospitals NHS Foundation Trust, Newcastle, NE1 3BZ, UK; 3 BHF Centre for Cardiovascular Science, University of Edinburgh, Midlothian, EH25 9RG, UK; 4 Institute of Ageing and Chronic Disease, University of Liverpool, Liverpool, L7 8TX, UK; 5 Developmental Endocrinology Research Group, School of Medicine, University of Glasgow, Glasgow, G51 4TF, UK

**Keywords:** Duchenne muscular dystrophy, Growth, Skeletal development, Marrow adiposity, Micro-CT, Growth plate

## Abstract

The muscular dystrophy X-linked (*mdx*) mouse is commonly used as a mouse model of Duchenne muscular dystrophy (DMD). Its phenotype is, however, mild, and other mouse models have been explored. The *mdx:Cmah^−/−^* mouse carries a human-like mutation in the *Cmah* gene and has a severe muscle phenotype, but its growth and bone development are unknown. In this study, we compared male *mdx*, *mdx:Utrn*^+/−^, *mdx:Cmah^−/−^* and wild-type (WT) mice at 3, 5 and 7 weeks of age to determine the suitability of the *mdx:Cmah^−/−^* mouse as a model for assessing growth and skeletal development in DMD. The *mdx:Cmah^−/−^* mice were lighter than WT mice at 3 weeks, but heavier at 7 weeks, and showed an increased growth rate at 5 weeks. Cortical bone fraction as assessed by micro-computed tomography was greater in both *mdx* and *mdx:Cmah^−/−^* mice versus WT mice at 7 weeks. Tissue mineral density was also higher in *mdx:Cmah^−/−^* mice at 3 and 7 weeks. Gene profiling of *mdx:Cmah^−/−^* bone identified increased expression of *Igf1*, *Igf1r* and *Vegfa*. Both the *mdx* and *mdx:Cmah^−/−^* mice showed an increased proportion of regulated bone marrow adipose tissue (BMAT) but a reduction in constitutive BMAT. The *mdx:Cmah^−/−^* mice show evidence of catch-up growth and more rapid bone development. This pattern does not mimic the typical DMD growth trajectory and therefore the utility of the *mdx:Cmah^−/−^* mouse for studying growth and skeletal development in DMD is limited. Further studies of this model may, however, shed light on the phenomenon of catch-up growth.

This article has an associated First Person interview with the first author of the paper.

## Abstract (precis)

**Summary:** Unlike boys with DMD, the *mdx:Cmah^−/−^* mouse shows increased weight gain and more rapid bone development; therefore, its utility for studying growth and skeletal development in DMD is limited.

## INTRODUCTION

Duchenne muscular dystrophy (DMD) affects 1 in 4000 live male births and is a severe and ultimately fatal X-linked recessive disease (Emery, 1991). Although progression of DMD can be slowed with the administration of glucocorticoids (GCs), therapy is often associated with growth retardation and skeletal fragility (Biggar et al., 2006; Joseph et al., 2019). However, it is clear that growth impairment and fractures are also prevalent in steroid-naïve boys with DMD (Rapaport et al., 1991; Larson and Henderson, 2000; Matsumoto et al., 2017), suggesting that there may be an intrinsic abnormality of growth and skeletal development in DMD (Eiholzer et al., 1988). Specifically, an older retrospective study that was carried out before GC therapy became standard of care found that 44% of steroid-naïve DMD boys had sustained a fracture (Larson and Henderson, 2000). A more recent *in vivo* and clinical study has also suggested that an abnormality of osteoblast function may exist in bones of DMD patients (Rufo et al., 2011). To improve our understanding of the underlying defect in growth and skeletal development in DMD, there is a critical need to identify an animal model that closely mimics these clinical features of DMD.

The muscular dystrophy X-linked (*mdx*) mouse is the most commonly used and best-characterised animal model of DMD, but it has limited applicability in humans as the muscle phenotype is much less severe (Bulfield et al., 1984; Muntoni et al., 1993). Acute muscle necrosis by 3 weeks of age is followed by subsequent muscle regeneration and the overall lifespan of the *mdx* mouse is nearly normal (Chamberlain et al., 2007). In contrast to the relentless decline in muscle function in humans, the muscle force of *mdx* limb muscles is near normal until the mice become aged (Lynch et al., 2001; Muller et al., 2001). The utrophin heterozygous *mdx* mouse (*mdx:Utrn^+/−^*) may represent an intermediate model between the extreme *mdx:Utrn* double-knockout mouse (*mdx:Utrn^−/−^*), which rarely survives beyond 20 weeks of age (Gao et al., 2019), and the more mildly affected *mdx* mouse. Utrophin is an autosomal homologue of dystrophin and its upregulation in the *mdx* mouse may compensate for the lack of dystrophin, thereby accounting for the less-severe phenotype compared to DMD in humans (Grady et al., 1997). However, the growth and skeletal development of the *mdx:Utrn^+/−^* mouse has not previously been studied in detail.

An alternative murine model of DMD is the *mdx:Cmah^−/−^* mouse. This carries a human-like mutation in the cytidine monophospho-N-acetylneuraminic acid hydroxylase (*Cmah*) gene that prevents synthesis of the sialic acid, N-glycolylneuraminic acid (Chou et al., 1998; Chandrasekharan et al., 2010). The *mdx:Cmah^−/−^* mouse has been reported to have phenotypic and molecular similarities to human DMD, and also displays increased disease severity and reduced lifespan compared to the *mdx* mouse (Chandrasekharan et al., 2010). The *mdx:Cmah*^−/−^ mouse may therefore be a more appropriate murine model for DMD, but its growth and skeletal phenotypes have not yet been characterised.

Bone marrow adipose tissue (BMAT) has attracted recent interest as a further biomarker of bone integrity and fracture risk, with increased BMAT often coinciding with decreased bone mass and increased skeletal fragility (Scheller et al., 2016). BMAT can be classified into two broad subtypes, regulated (rBMAT) and constitutive (cBMAT), with the former typically showing greater increases in conditions of bone fragility (Scheller et al., 2016). There is also evidence to show that muscle disuse is associated with an increase in BMAT (Gorgey et al., 2013). However, the impact of DMD on BMAT has not been assessed previously.

The aim of this study was to further establish and compare the bone structure and function, BMAT quantity, and growth phenotypes of the *mdx*, *mdx:Utrn^+/−^* and *mdx:Cmah^−/−^* DMD mouse models with the C57BL/10 wild-type (WT) mouse, and determine which model would most closely mimic the clinical characteristics of DMD.

We hypothesised that growth, growth plate (GP) chondrogenesis and bone development are impaired in the muscular dystrophy mouse models when compared to WT mice, and more severely in the *mdx:Cmah^−/−^* mouse model. In addition, rBMAT, but not cBMAT, would be inversely associated with bone loss in each model. Accurate characterisation of bone and growth would enable the selection of an appropriate animal model when testing new therapies.

## RESULTS

### Grip strength and inflammation in muscular dystrophy models

**Fig. 1.**
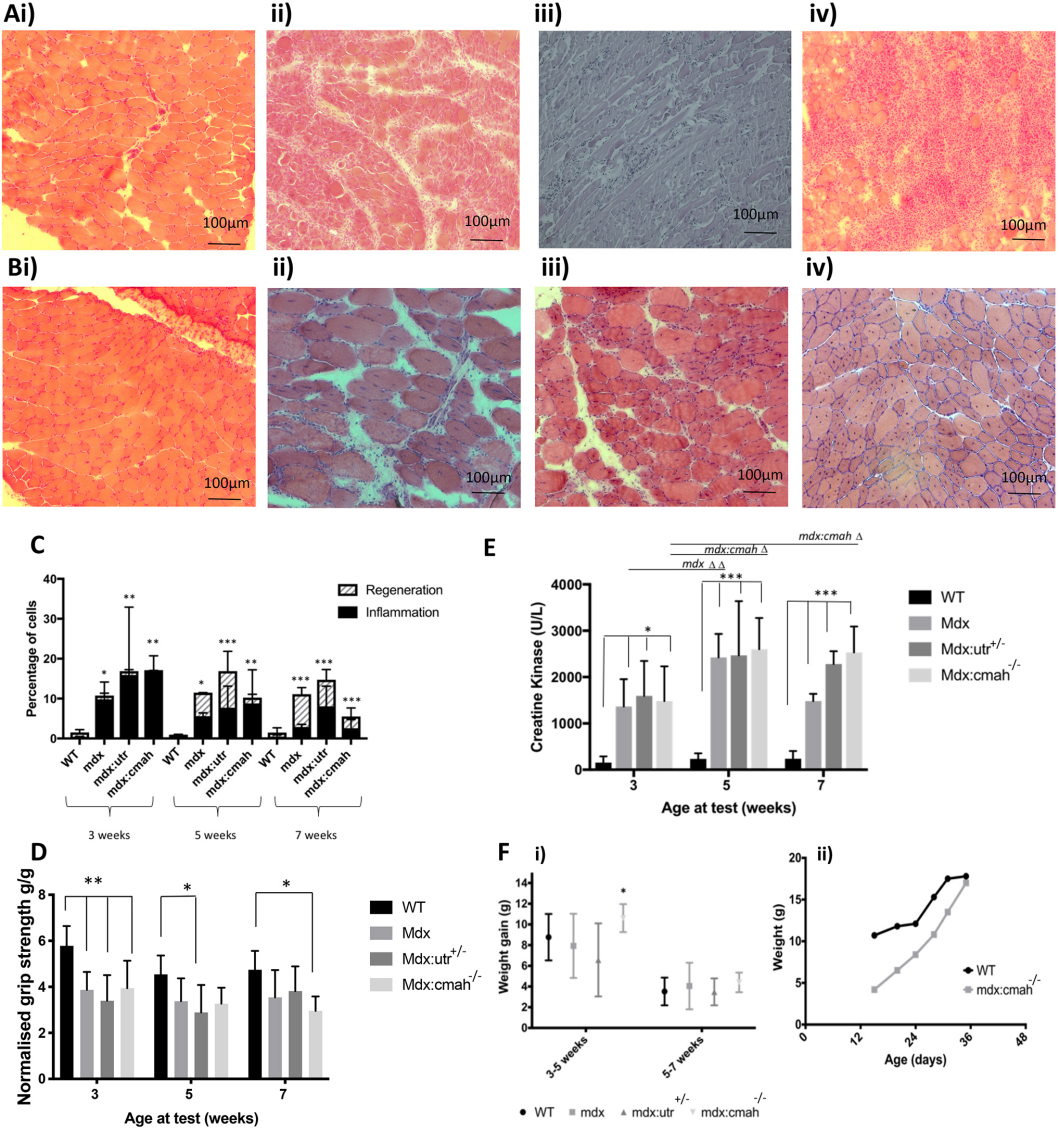
**Muscle characteristics in different muscular dystrophy mouse models.** (A) H&E-stained section of tibialis anterior from a 3-week-old: (i) WT mouse showing normal, regular myofibres with peripheral nuclei and intact sarcoplasm; and (ii) *mdx*, (iii) *mdx:Utrn^+/−^* (shown as mdx:utr in the figure) and (iv) *mdx:Cmah^−/−^* mouse, showing many inflammatory cells with a barely visible sarcoplasm. (B) H&E-stained section of tibialis anterior from a 7-week-old: (i) WT mouse showing normal, regular myofibres with peripheral nuclei and intact sarcoplasm; and (ii) *mdx*, (iii) *mdx:Utrn^+/−^* and (iv) *mdx:Cmah^−/−^* mouse, showing regeneration with larger myofibres and central nuclei. (C) Muscle cell inflammation was present in all muscular dystrophy models by 3 weeks of age followed by regeneration at 5 and 7 weeks of age. (D) Mean grip strength by age and genotype, showing a reduction in muscular dystrophy mice. (E) Higher CK activity in muscular dystrophy mice at all ages. Data are presented as mean±s.d. (*n*>6); *P<0.05, **P<0.01, ***P<0.001 for cumulative measures of muscle damage compared to WT mice. (F) Increased weight gain is seen in the young *mdx:Cmah^−/−^* mice. (Fi) Increased rate of weight gain in the *mdx:Cmah^−/−^* mice occurred between 3 and 5 weeks of age but not between 5 and 7 weeks of age. Data presented are mean (symbol) and standard deviation (whiskers). *P<0.05 compared to WT mice. (Fii) Example of a growth chart for a WT mouse compared to an *mdx:Cmah^−/−^* mouse, showing the lower initial weight and the rapid growth velocity seen in the early weeks in the *mdx:Cmah^−/−^* mouse*.*

Histology of the tibialis anterior muscle revealed areas of necrosis with a significant increase in the percentage of inflammatory cells within all the muscular dystrophy models at 3 weeks of age (Fig. 1A). There was evidence of muscle fibre necrosis and regeneration with an increase in centrally located myonuclei and fibre size variation by 5 and 7 weeks of age, which was particularly noticeable in the *mdx* and *mdx:Utrn*^+/−^ mice (Fig. 1Bii,iii). This is compared to WT mice, whose muscle histology showed normal, regular myofibres with peripheral nuclei and intact sarcoplasm at all ages studied, with minimal evidence of inflammation or regeneration (Fig. 1Ai,Bi,C). Grip strength (normalised to body weight) was less in all muscular dystrophy models at 3 weeks of age compared to WT mice (*P*<0.01; Fig. 1D). Reduced grip strength was also noted in all muscular dystrophy models at 5 and 7 weeks of age but the changes did not always reach significance (Fig. 1D). Serum creatine kinase (CK) activity was >10-fold higher in all muscular dystrophy mice and at all ages compared to WT mice, providing further evidence for muscle damage in these muscular dystrophy models (Fig. 1E).

### Anthropometric measurements

Body weight and body length were measured from weaning (3 weeks of age) (Table 1). No differences were observed in the body weight of the *mdx* or *mdx:Utrn*^+/−^ mice compared to WT at any time point. In contrast, *mdx:Cmah^−/−^* mice were almost 2 g lighter than WT mice at 3 weeks of age (8.07 g versus 10.03 g, *P*=0.02), but were 3 g heavier by 7 weeks of age (21.97 g versus 24.97 g, *P*=0.02). Consistent with this, when analysing the mice that were culled at 5 or 7 weeks of age (i.e. those with longitudinal growth data available), the *mdx:Cmah^−/−^* mice gained significantly more weight than WT mice from 3 to 5 weeks of age (8.77 g versus 10.61 g, *P*=0.01). Weight gain slowed between 5 and 7 weeks of age in all models, and there were no genotype differences in weight gain during this period (Fig. 1F). When genotype and age were incorporated into a multivariable linear regression model, *mdx:Cmah^−/−^* mice were associated with an increased growth rate [coefficient (coeff) 1.84, *P*<0.05] during the overall study period. No differences in body weight were observed in the *mdx* or *mdx:Utrn*^+/−^ mice compared to WT mice at any time point studied.

**Table 1.**
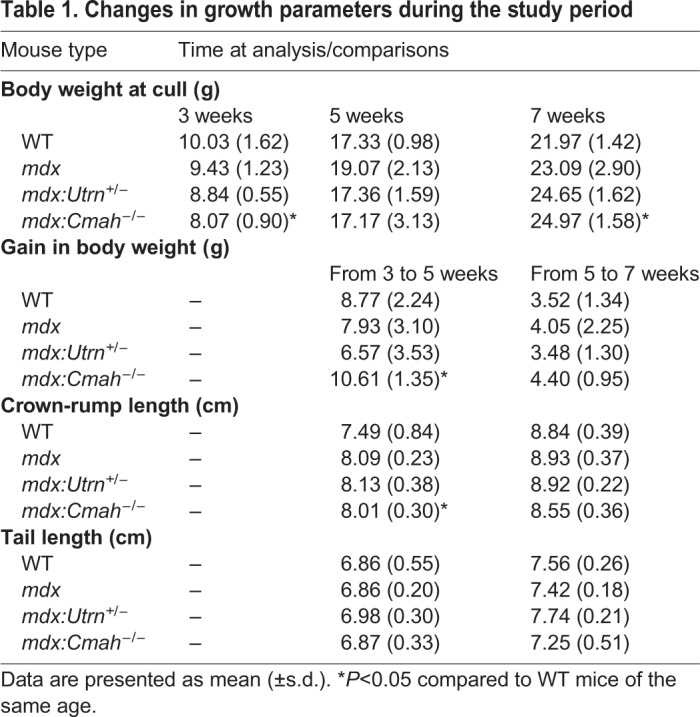
**Changes in growth parameters during the study period**

### Tibia length, growth rate and growth plate analysis

**Fig. 2.**
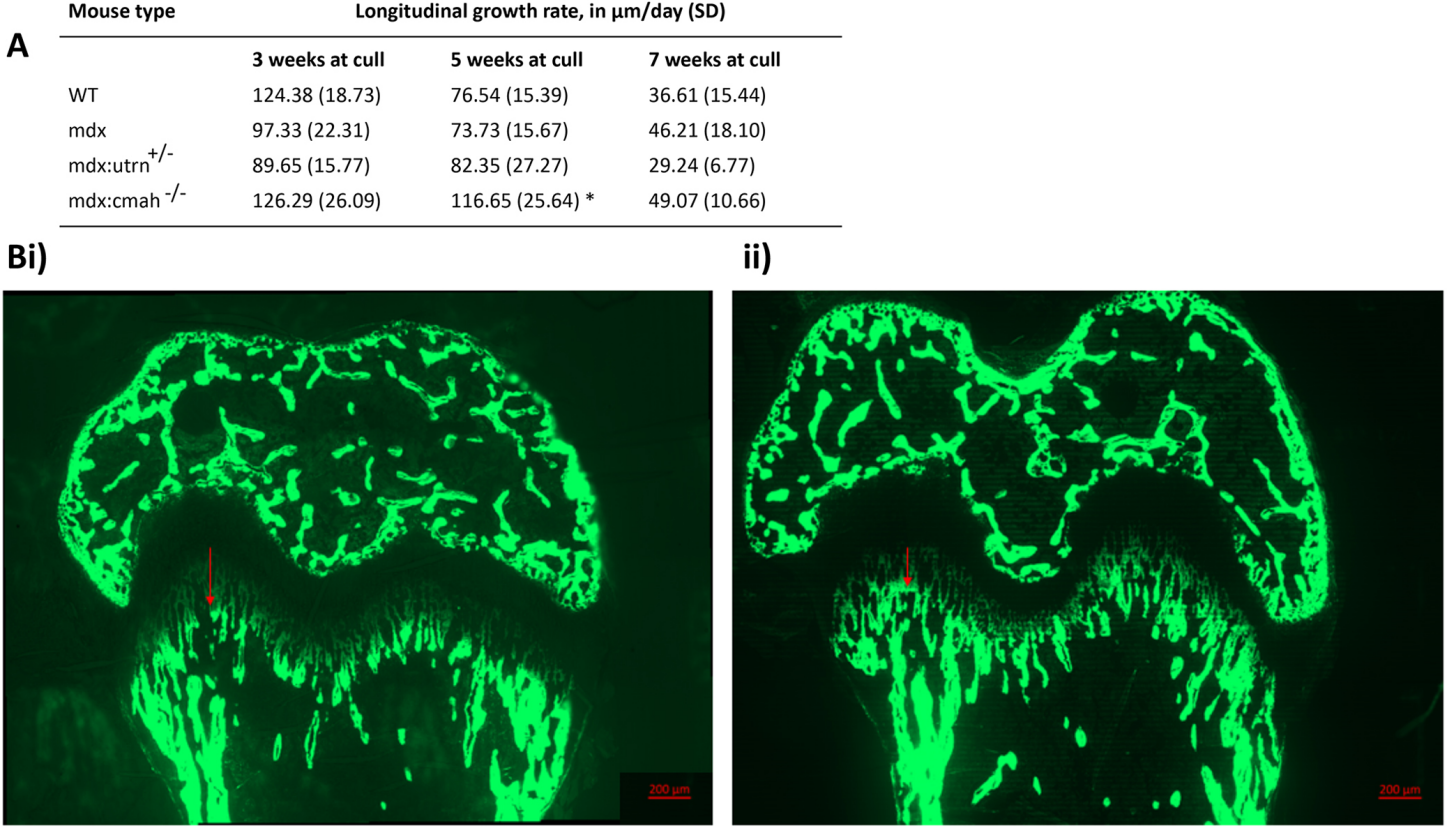
**Longitudinal bone growth rate in muscular dystrophy mouse models.** (A) Longitudinal bone growth during the study period. Data are presented as mean±s.d. *P<0.05 compared to WT mice of the same age. (B) Examples of calcein-labelled GP in a 5-week-old (i) *mdx:Cmah^−/−^* and (ii) *mdx* mouse, showing the increased longitudinal growth rate in the *mdx:Cmah^−/−^* mouse (red arrows).

Tibia length from similarly aged WT and muscular dystrophy mice was comparable, as was the rate of longitudinal bone growth (assessed by calcein labelling); one exception was for *mdx:Cmah^−/−^* mice, which had an increased growth rate at 5 weeks of age (*P*=0.05; Fig. 2). When genotype and age at cull were incorporated into a multivariable linear regression model, *mdx:Cmah^−/−^* mice were associated with an increased overall longitudinal bone growth rate within the GP (coeff 18.6, *P*<0.01) compared with WT and the other muscular dystrophy mouse models. Closer examination of the proximal tibial GP of 3- and 7-week-old muscular dystrophy mice also revealed no changes in total GP width or the width of the proliferative and hypertrophic zones (Fig. S1). Similarly, chondrocyte proliferation was unaffected in all of the muscular dystrophy mice at 3 weeks of age (Fig. S1). We attempted to assess the number of apoptotic hypertrophic chondrocytes in 7-week-old mice, but the numbers of these cells were too sparse to enable accurate quantification (data not shown).

### Bone phenotype in the muscular dystrophy models

**Fig. 3.**
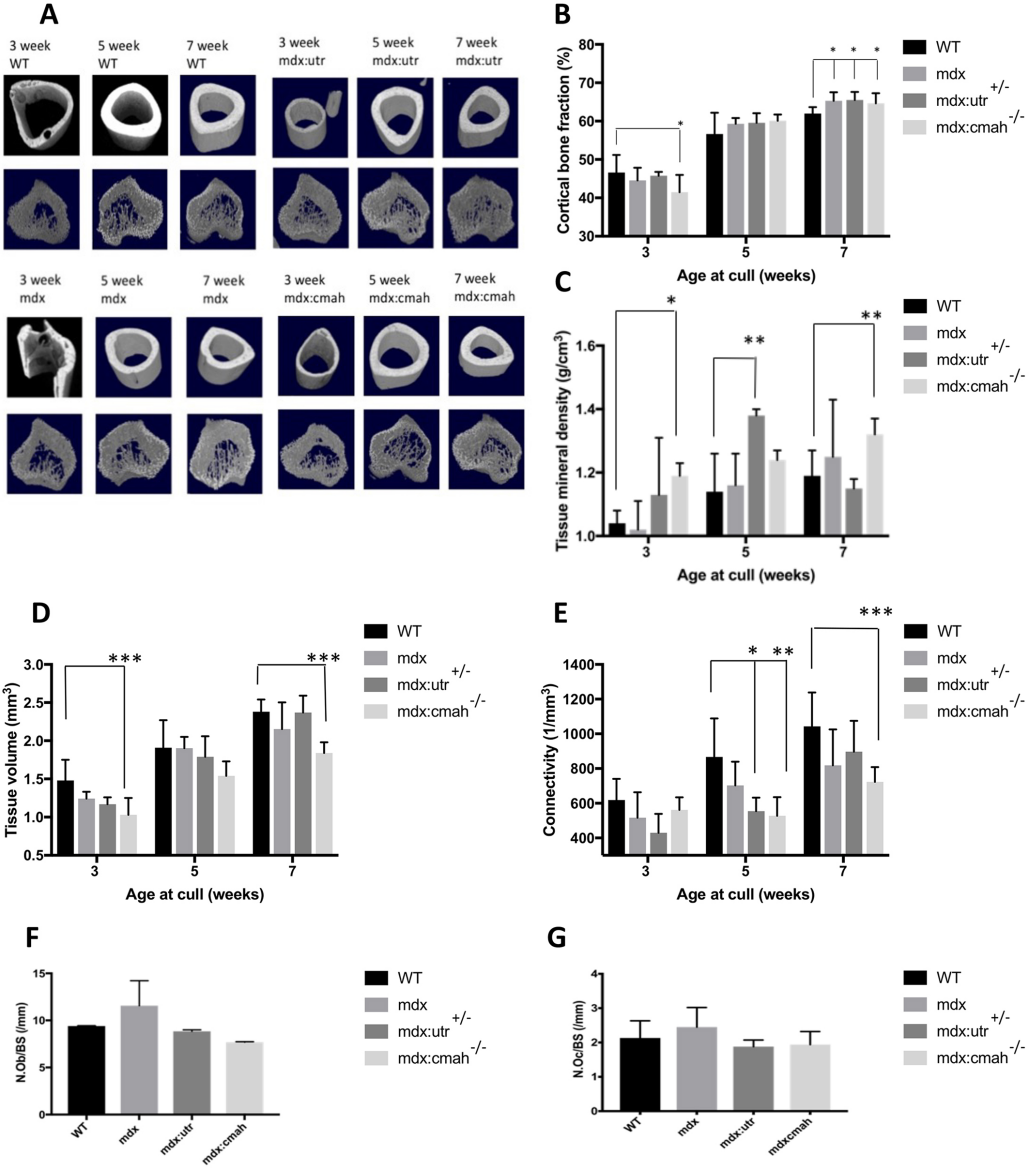
**Bone parameters in muscular dystrophy mouse models.** (A) Representative µCT images of tibial bone of all genotypes at 3, 5 and 7 weeks of age to show bone development (upper row, mid diaphyseal cortical bone; lower row, metaphyseal trabecular bone). (B) Compared to similarly aged WT mice, cortical bone fraction was lower in *mdx:Cmah**^−^******^/−^*** mice at 3 weeks of age, but higher in all muscular dystrophy models at 7 weeks of age. (C) Cortical tissue mineral density was higher in *mdx:Cmah**^−^******^/−^*** mice at 3 and 7 weeks of age and in *mdx:Utrn^+/−^* (shown as mdx:utr in the figure) mice at 5 weeks when compared to WT mice. (D) Trabecular tissue volume was lower in *mdx:Cmah**^−^******^/−^*** mice at 3 and 7 weeks of age compared with WT. (E) Trabecular connectivity was lower in *mdx:Utrn^+/−^* mice at 5 weeks and *mdx:Cmah**^−^******^/−^*** mice at both 5 and 7 weeks when compared to WT mice. (F,G) The number of osteoblasts (F; N.Ob/BS) and osteoclasts (G; N.Oc/BS) per bone surface were normal in all muscular dystrophy models at 5 weeks of age. Data are presented are mean±s.d. (*n*>6); *P<0.05, **P<0.01, ***P<0.001 compared to WT mice.

Very few structural changes were observed in the cortical bone of the muscular dystrophy mice compared to WT mice (Table 2, Table S1, Fig. 3A). Cortical bone area and cortical bone fraction were lower in *mdx:Cmah^−/−^* mice compared to WT mice at 3 weeks of age (*P*<0.05). However, in all 7-week-old muscular dystrophy models, a higher cortical bone fraction was noted compared to WT mice (*P*<0.05; Fig. 3B). Cortical tissue mineral density (TMD) was higher in *mdx:Cmah^−/−^* mice at 3 (*P*<0.05) and 7 (*P*<0.01) weeks of age and in *mdx:Utrn*^+/−^ mice at 5 weeks (*P*<0.01) when compared to WT mice (Fig. 3C). Trabecular tissue architecture was also relatively unchanged in the various muscular dystrophy models (Table 2, Table S2, Fig. 3A). The most stark difference was in trabecular tissue volume, which was lower in *mdx:Cmah^−/−^* mice at all ages studied, and this reached significance at 3 and 7 weeks of age (*P*<0.001; Fig. 3D). Also, there was a trend for lower trabecular connectivity in all muscular dystrophy mice, and this reached significance in the *mdx:Utrn*^+/−^ mice at 5 weeks (*P*<0.05) and *mdx:Cmah^−/−^* mice at both 5 and 7 weeks (*P*<0.01) when compared to WT mice (Fig. 3E). The lack of a major bone phenotype was reflected by the normal number of osteoblasts and osteoclasts present on trabecular bone surfaces of the muscular dystrophy models (Fig. 3F,G). Serum markers of bone formation and resorption were also relatively normal in muscular dystrophy mice, although αCTX concentrations were significantly higher in 3- to 5-week-old *mdx:Cmah^−/−^* mice (406.36±46.51 pg/ml) compared to WT controls (181.70±78.51 pg/ml; *P*<0.01).

**Table 2.**
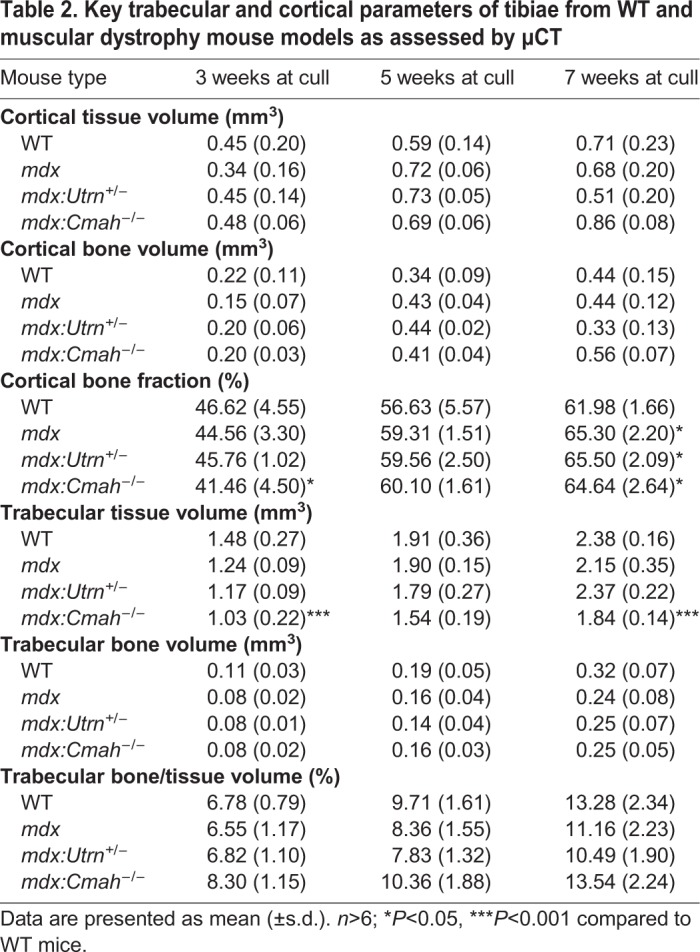
**Key trabecular and cortical parameters of tibiae from WT and muscular dystrophy mouse models as assessed by µCT**

### Biomechanical properties

Consistent with the small structural changes observed in the trabecular and cortical compartments, there were limited differences in the biomechanical properties of bones from WT and muscular dystrophy mice. The only exception was a trend for lower deflection at maximum load in *mdx:Cmah^−/−^* mice at all ages studied, and this difference was significant in mice at 5 weeks of age (Table 3).

**Table 3.**
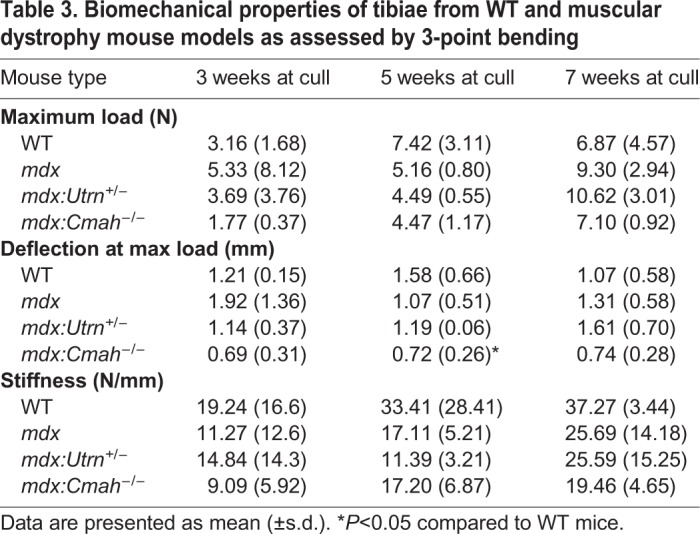
**Biomechanical properties of tibiae from WT and muscular dystrophy mouse models as assessed by 3-point bending**

### Gene and protein expression studies

To identify more subtle changes in diaphyseal bone from muscular dystrophy mice, we next used a PCR array to profile the expression of 84 genes involved in skeletal development and/or bone mineral metabolism. This analysis was limited to bone from 7-week-old mice and revealed a 2- to 3.75-fold upregulation of matrix metalloprotease 10 (*Mmp10*) and bone morphogenetic protein receptor type 1b (*Bmpr1b*) in all muscular dystrophy models compared to WT mice (Table 4). In *mdx:Cmah^−/−^* mice, there was also increased expression of growth and transcription factors compared to WT control mice. In particular, there was increased expression of insulin-like growth factor-1 (*Igf1*; 30-fold), IGF-1 receptor (*Igf1R*; 10-fold) and vascular endothelial growth factor A (*Vegfa*; 32-fold) (Table 4).

**Table 4.**
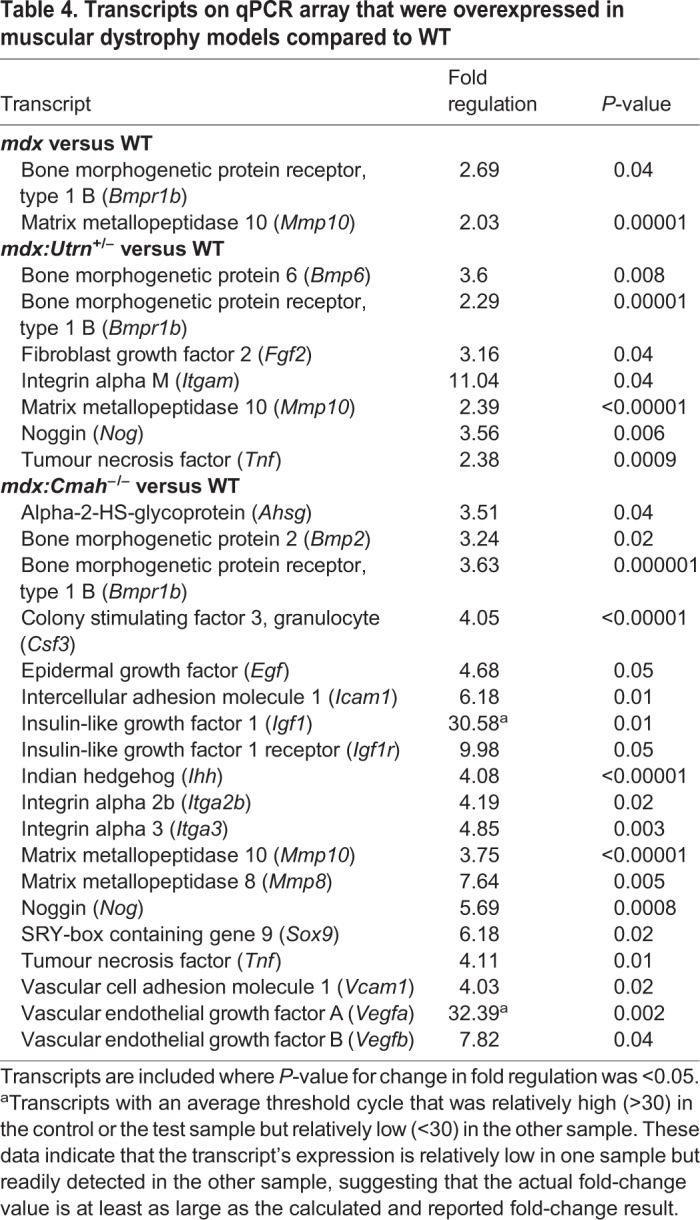
**Transcripts on qPCR array that were overexpressed in muscular dystrophy models compared to WT**

**Fig. 4.**
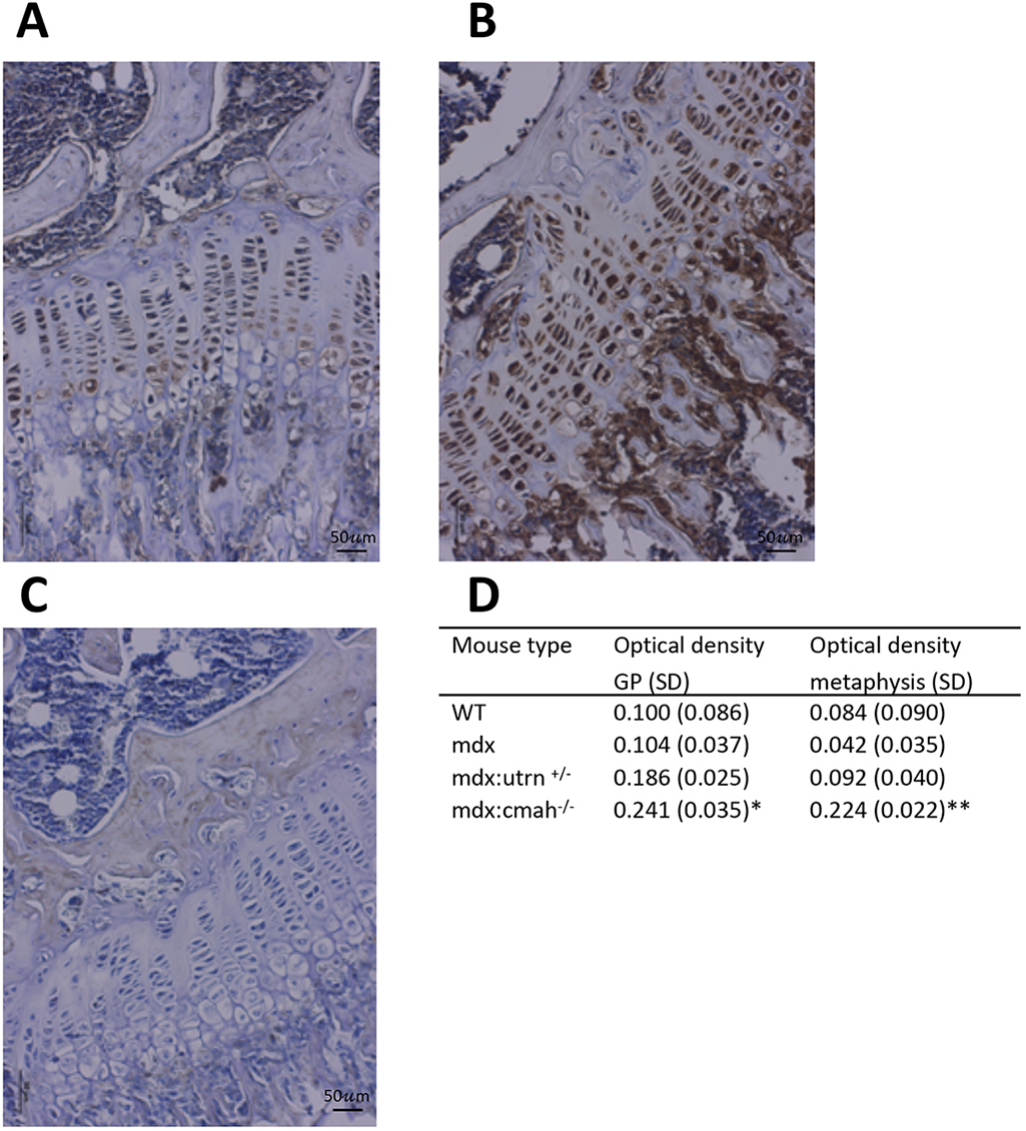
**IGF-1 immunohistochemistry in muscular dystrophy mouse models.** Immunolocalisation of IGF-1 in sections of (A) WT and (B) *mdx:Cmah^−/−^* tibiae, showing greater staining intensity in cells of both ossification centres and the growth plate (GP). (C) Control section in which rabbit IgG was substituted for the primary antibody. (D) Quantification of IGF-1 staining in GP chondrocytes and metaphysis of tibiae from WT and muscular dystrophy mouse models. Data are presented as mean±s.d. (*n*=6). *P<0.05, **P<0.01 compared to WT mice.

Selected genes from this array were validated by immunohistochemistry (IHC) to confirm the presence of IGF-1, BMPR1b and MMP-10 proteins in tibial sections from WT and muscular dystrophy mice. MMP-10 and BMPR1b were chosen because they were upregulated in all muscular dystrophy mouse models. IGF-1 was chosen because it is an essential factor in the regulation of postnatal growth and showed a marked upregulation in bone of *mdx:Cmah^−/−^* compared to WT mice. We were unable to detect robust, positive staining for BMPR1b (data not shown). In contrast, MMP-10 immunolocalisation was successful, but was similar in all sections examined and no genotype-specific differences in staining distribution or intensity were noted (data not shown). IHC revealed greater IGF-1 staining of GP chondrocytes and cells associated with trabecular bone within the metaphysis (Fig. 4A-C). When quantified, IGF-1 staining intensity was significantly greater in sections of *mdx:Cmah^−/−^* mice compared to WT mice (Fig. 4D).

### BMAT quantification in tibiae

**Fig. 5.**
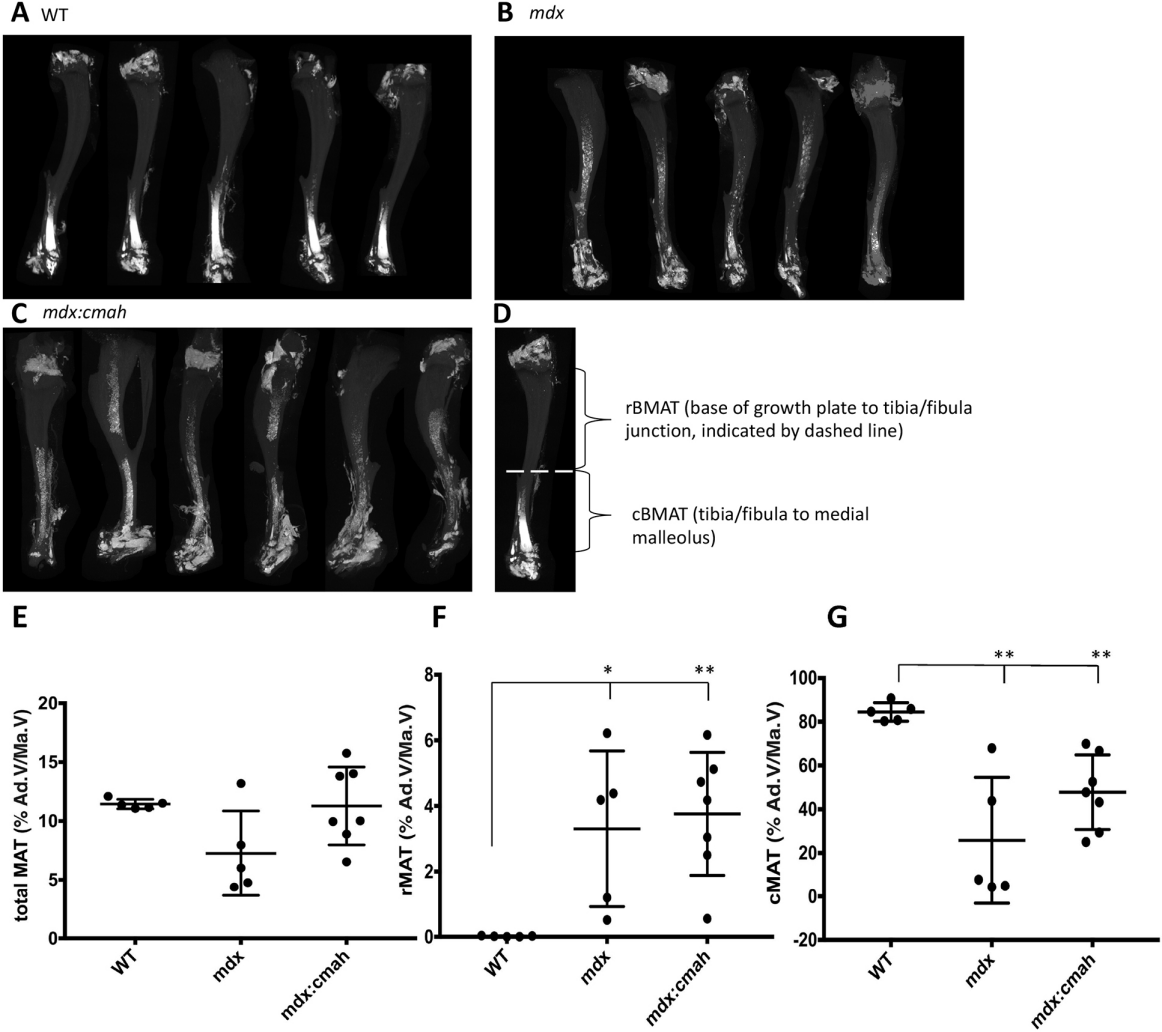
**Bone marrow adiposity in muscular dystrophy mouse models.** Three-dimensional µCT reconstructions of 7-week-old osmium-stained tibiae from: (A) WT, (B) *mdx* and (C) *mdx:Cmah^−/−^* mice. Bone appears grey and BMAT is white. (D) The boundaries of rBMAT and cBMAT within the tibia are shown; rBMAT lies proximal to tibia/fibula junction and cBMAT lies distal to tibia/fibula junction (shown by dashed line). (E-G) Percentage of (E) total BMAT, (F) rBMAT, (G) cBMAT. Data are presented as mean±s.d. (*n*=5). *P<0.05, **P<0.01 compared to WT mice. Ad.V/Ma.V, adipose volume/marrow volume.

The proportion of total BMAT within the tibial marrow cavity of WT and muscular dystrophy mice was similar. However, in comparison to WT mice, the percentage of rBMAT (% rBMAT) was higher in both *mdx* and *mdx:Cmah^−/−^* mice, whereas % cBMAT was lower in both muscular dystrophy models (Fig. 5). Although the BMAT of *mdx:Utrn*^+/−^ mice was not studied, these observations suggest that BMAT volume and distribution may be altered in DMD.

### Metatarsal culture

To determine whether there was an intrinsic skeletal insult in these muscular dystrophy models, we also investigated the growth potential of bones from WT, *mdx* and *mdx:Cmah^−/−^* mice in an *ex vivo* organ culture model. Over a 7-day period, the growth of metatarsals from muscular dystrophy and WT mice was similar (Fig. S1D).

## DISCUSSION

We have demonstrated a significant muscle phenotype in all three muscular dystrophy models, and also confirmed age- and strain-related characteristics. Consistent with published data, the *mdx:Cmah^−/−^* mice exhibited a marked muscle phenotype (Chandrasekharan et al., 2010). This is consistent with the theory that lower levels and function of the dystrophin-glycoprotein complex and metabolic accumulation of dietary N-glycolylneuraminic acid, which occur as a consequence of the *Cmah* mutation, contribute to a more-severe muscle phenotype. Indeed, our analyses of muscle histology and serum CK further demonstrate that *mdx* mice have a greater capacity for muscle regeneration than the *mdx:Utrn*^+/−^ or *mdx:Cmah^−/−^* models. In keeping with this, normalised grip strength was significantly lower in *mdx* compared to WT at 3 weeks of age, but not beyond this time point. This is consistent with published data on the *mdx* mouse suggesting that there is an early period, between the end of the second and up to the fifth week of life, when the mice experience histological necrosis and functional weakness. After this critical period, regeneration occurs and there are no co-existing clinical features of DMD, such as scoliosis and heart failure, until mice are at least 15 months or older (Bulfield et al., 1984; Muntoni et al., 1993; Lefaucheur and Sebille, 1995; Chamberlain et al., 2007; Coley et al., 2016). Like all the muscular dystrophy models that we tested, serum CK activity is significantly higher in *mdx:Utrn*^+/−^ mice than WT mice, and muscle histology also demonstrated ongoing inflammation and regeneration at 5 and 7 weeks of age. However, the normalised grip strength was lower than WT mice only at 3 weeks of age. Other studies have also reported a variable muscle phenotype (Huang et al., 2011; Mcdonald et al., 2015).

Our study is the first to describe the growth and bone phenotypes of the *mdx:Cmah^−/−^* and *mdx:Utrn^+/−^* mouse. It was expected that there would be a growth phenotype in all the muscular dystrophy models and that it would be most severe in the *mdx:Cmah^−/−^* mice. However, our results do not support this and, despite the marked muscle phenotype, we find no evidence of a more severe skeletal phenotype in mice of up to 7 weeks of age. Unexpectedly, despite being bred in exactly the same conditions and during the same season, the *mdx:Cmah^−/−^* mice are smaller than the WT mice at 3 weeks of age, but demonstrated increased weight gain such that, by 7 weeks of age, they are heavier than WT mice. The smaller absolute body weight in the youngest *mdx:Cmah^−/−^* mice may be a reflection of several factors, including poorer general health in the mothers prior to or during pregnancy, or a problem pre- or peri-weaning in the offspring. Consistent with this, the *mdx:Cmah^−/−^* mice were much harder to breed than expected and there were a higher than expected number of pre-wean deaths. The catch-up growth is suggested by gross body weight measures but also at the GP level through dynamic histomorphometry using calcein labelling. Additional evidence for the catch-up growth is found in the upregulation of many growth factors on the PCR array, including a 30-fold increase in *Igf1* expression and 60-fold increase in *Vegfa* expression*.* Further evidence of increased bone turnover in the young *mdx:Cmah^−/−^* mice compared to WT mice is suggested by the higher levels of α-CTX, a marker of bone resorption. Although levels of the bone formation marker, P1NP, were not raised at this age, it is possible that it may have risen subsequently.

We did not find any evidence of growth retardation in either the *mdx* or *mdx:Utrn*^+/−^ mice compared to WT mice at any age. Consistent with our data showing no difference in tibial length (Table S3), Nakagaki showed no significant difference in femur length between *mdx* and WT mice at 3 week of age (Nakagaki et al., 2011).

There are conflicting data regarding the bone phenotype in the *mdx* mouse and, unlike in patients with DMD, where bone strength decreases with advancing age, the converse appears true in some studies of *mdx* mice (Anderson et al., 1993). Osteopenia has also been demonstrated in young *mdx* mice (Anderson et al., 1993; Nakagaki et al., 2011; Novotny et al., 2011). By contrast, other studies have shown either no difference in any µCT parameters between young *mdx* and WT males, or a paradoxical increase in femoral bone mineral density on dual-energy X-ray absorptiometry in older female *mdx* mice compared to WT mice (Montgomery et al., 2005; Isaac et al., 2013). We have shown a higher cortical bone fraction in *mdx* mice compared to WT mice, but only at 7 weeks of age, and no difference was observed in any other parameters on micro-computed tomography (µCT), bone turnover markers or on biomechanical testing. This may reflect the muscle-bone crosstalk and the potential for an adaptive bone response alongside the muscle regeneration in the *mdx* model.

Compared to the *mdx* mouse, data reporting on the bone structure of *mdx:Utrn*^+/−^ mice is restricted to collagen content of the metaphyseal bone, which is less in *mdx:Utrn*^+/−^ mice compared to *mdx* and WT mice (Isaac et al., 2013). This present study also shows no conclusive evidence of altered bone structure in *mdx:Utrn*^+/−^ mice. Our µCT data show, paradoxically, that TMD is higher in the *mdx:Utrn*^+/−^ mice compared to WT mice at 5 weeks of age only, while cortical bone fraction is also greater than WT mice, but only in those culled at 7 weeks.

Although we hypothesised that *mdx:Cmah^−/−^* mice would have osteoporosis, their TMD is increased compared to WT mice at both 3 and 7 weeks of age, and cortical bone fraction is also higher at 7 weeks of age. This may be due to a compensatory bone response to muscle damage and then regeneration as described for *mdx* mice, but there may also be other factors responsible. For example, one study demonstrated that the *CMAH* gene is upregulated in adult human stem cells, of both haematopoietic and mesenchymal origin. Overexpression of the human *CMAH* gene causes accumulation of nuclear β-catenin (Nystedt et al., 2009) and associated restriction of Wnt signalling. Therefore, it is possible that the absence of the *Cmah* gene in the *mdx:Cmah^−/−^* mouse model may cause a reduction in nuclear β-catenin expression and unregulated Wnt signalling, and thereby promote the osteoblast lineage and bone formation (Westendorf et al., 2004).

We found that all the muscular dystrophy models have an upregulation of *Mmp10* and *Bmpr1b* gene expression compared to WT mice. Matrix metalloproteases (MMPs) are members of a family of calcium-dependent proteinases that can cleave a wide range of extracellular proteins, including components of the extracellular matrix. MMP-10 is known to be involved in bone growth but has also been shown to be involved in tissue repair processes and specifically in muscular dystrophy mouse models (Ortega et al., 2004). Increased skeletal muscle MMP-10 protein expression has been demonstrated in response to the damaged, dystrophic muscle seen in the *mdx* mouse (Ortega et al., 2004). This may also explain our finding that *Mmp10* is upregulated in bone, because the bone-muscle unit is intrinsically interlinked (Veilleux and Rauch, 2017). Growth factors of the transforming growth factor (TGFβ) family, which includes bone morphogenetic proteins (BMPs), play an important role in skeletal development (Chen et al., 2012), and BMP signalling in particular has been shown to lead to the expression and activity of genes necessary for osteoblast differentiation (Song et al., 2009). Increased BMP signalling has also been implicated in the pathogenesis of DMD, and recent studies have suggested an essential role of BMPs and also the type 1 receptor in regeneration after muscle damage (Shi et al., 2013). Thus, our findings of increased skeletal *Bmpr1b* expression in *mdx*, *mdx:Utrn*^+/−^ and *mdx:Cmah^−/−^* mice are consistent with their use as preclinical muscular dystrophy models.

Our finding of catch-up growth in *mdx:Cmah^−/−^* mice has further implications for understanding the putative role of *Cmah* in other diseases. Thus, *Cmah*-null mice have been proposed as one of the best to mimic the phenotype of human metabolic disorders (Kavaler et al., 2011; Kwon et al., 2015). We speculate that this metabolic dysregulation may relate to altered developmental programming in *Cmah-*null mice. According to the Barker hypothesis and the developmental origins of health and disease, predisposition to the development of the metabolic syndrome begins *in utero* during critical periods of foetal development. During such periods, changes occur to promote foetal survival in the adverse intrauterine environment, but, in later life, these changes can also have a lasting impact on health and the development of the metabolic syndrome. The *mdx:Cmah^−/−^* mice in this study were smaller than expected for the C57BL/10 background and displayed catch-up growth, in keeping with the ‘thrifty phenotype’, but the process and the exact role of the *Cmah* mutation remains unclear. One possibility is that these mice have mitochondrial dysfunction caused by oxidative stress, but it is unclear whether this is a cause or consequence of their insulin resistance (Kwon et al., 2015).

The assessment of BMAT provided some very interesting and unexpected findings. We have shown that there is a significant increase in rBMAT in both the *mdx* and *mdx:Cmah^−/−^* mice, but a marked decrease in cBMAT. The causes and consequences of this increase, as well as its clinical relevance, warrant further investigation. Several mechanisms might account for the altered BMAT. Studies have shown that rBMAT is increased during obesity and, paradoxically, also during caloric restriction (Devlin, 2011; Cawthorn et al., 2014), whereas loss of cBMAT is less common and is most pronounced in lipodystrophic conditions. This indicates modulation of BMAT in states of altered systemic energy homeostasis. The role of dystrophin in the regulation of metabolism is poorly understood and is difficult to evaluate clinically as body composition in DMD is confounded by the long-term use of high-dose GCs. However, a recent study demonstrated that dystrophin deficiency in the *mdx* mouse resulted in reduced body fat (confirmed by DEXA body composition studies) and a consistently lower core body temperature, despite no overall body weight difference between the *mdx* and WT mice (Strakova et al., 2018). Similar studies have shown increases in energy expenditure and muscle protein synthesis in the *mdx* mouse, and an altered response to high-fat diet compared to C57BL/10 controls (Radley-Crabb et al., 2011, 2014). The differences were found in spite of a higher level of food consumption. Thus, it is possible that the increase in rBMAT occurs as a response to altered energy homeostasis in the DMD mice. In humans, increased BMAT is associated with increased plasma lipids (Scheller et al., 2016); however, studies examining serum lipid levels have shown no difference between *mdx* and WT mice (Milad et al., 2017). In contrast, *mdx* mice appear to have defects in expansion of primary adipose progenitor cells *in vitro* (Joseph et al., 2018), and therefore altered BMAT may also relate to differences in adipose progenitor cells.

The consequences of altered BMAT are also interesting to consider. Marrow adipocytes have been suggested to regulate skeletal remodelling, haematopoiesis and metabolic homeostasis. Moreover, logic dictates that accumulation of rBMAT in proximal tibia must occur at the expense of either haematopoiesis or bone, given the finite size of the marrow cavity. Consistent with this, we found that trabecular bone is decreased in 7-week-old *mdx:Cmah^−/−^* mice. However, cortical bone in each muscular dystrophy model was increased at 7 weeks of age, which is at odds with the increased rBMAT. It would be worth assessing haematopoiesis in these mice, given the potential for BMAT to modulate this process. Clinically, osteoporosis has been associated with a decrease in BMAT unsaturation (Yeung et al., 2005) and bone-marrow fat composition has been suggested as a useful biomarker in post-menopausal women with fragility fractures (Patsch et al., 2013). Thus, altered BMAT may also have a useful prognostic role in DMD. Future studies should explore these possibilities.

### Conclusion

In conclusion, our results confirm that all three of the DMD mouse models examined showed marked muscle damage and impaired grip strength as expected and consistent with the literature. We have also demonstrated differences in BMAT composition, which may serve as a potential biomarker for bone fragility in patients with DMD. However, we find no consistent bone or growth defect in any of the DMD models at all ages studied. This may become apparent in older mice, but it is likely that, to mimic more closely the osteoporosis and growth retardation that are observed in boys with DMD, glucocorticoids must be given to the mice. In particular, we find that the *mdx:Cmah^−/−^* mouse certainly does not mimic the growth trajectory of boys with DMD, but instead appears to display catch-up growth and increased bone turnover with a resultant increase in TMD and cortical bone fraction. Rather than being a useful murine model for growth retardation and osteoporosis in DMD, the *mdx:Cmah^−/−^* mouse may in fact be useful when a model of catch-up growth is desired.

## MATERIALS AND METHODS

### Animals and experimental procedures

Four different mouse strains were examined: wild-type (WT; C57BL/10ScSnJ); *mdx* (C57BL/10ScSn.mdx); *mdx:Utrn* (C57BL/10ScSn.utrn/mdx); and *mdx:Cmah^−/−^* (B10.Cg-CMah^tm1Avrk^Dmdmdx/PtmJ). All mice were obtained from Jackson Laboratories (New Harbour, MA, USA). All animal experiments were approved by the Roslin Institute's Animal Users Committee, and mice were maintained in accordance with Home Office (UK) guidelines for the care and use of laboratory animals. All mice were housed under controlled temperature (approx. 25°C) and light conditions (12 h light:12 h dark cycle), and had access to food and water *ad libitum*. A cross-sectional study was performed whereby mice were culled at three developmental ages (3, 5 and 7 weeks of age). A minimum of 6 male mice of each genotype were used at each time point. Additional mice were used for the assessment of marrow adiposity. Body weight, crown-to-rump and tail-length measurements were taken twice weekly using digital weighing scales and a ruler.

### Grip strength

Forelimb grip strength testing was performed within 24 h prior to culling, using a grip strength meter with a specialised mouse grid (Harvard Biosciences, MA, USA), according to the TREAT-NMD standard operating protocol (Luca, 2014). The mean of three consecutive measurements was calculated and normalised to body mass.

### Muscle histology

The tibialis anterior muscles were removed and fixed in 4% paraformaldehyde (PFA) before undergoing paraffin wax embedding. Sections (6 µm) were stained with Haematoxylin and Eosin (H&E) for histological assessment, according to the TREAT-NMD protocol (Grounds, 2014). Images were acquired using a Zeiss AxioImager brightfield microscope and analysed using Fiji (Schindelin et al., 2012). The percentage of inflammatory cells in the region of interest (ROI) was calculated and the number of central nuclei (signifying muscle regeneration) recorded, to obtain a cumulative measure of skeletal muscle damage.

### Serum measurements

Immediately following euthanasia, blood was obtained from non-fasted animals via cardiac puncture. Serum CK concentration was measured by a CK assay kit (Pointe Scientific, Stroud, UK) in samples collected at 3, 5 and 7 weeks of age. Serum P1NP and αCTX was measured by ELISA (AMS Biotechnology, Abingdon, UK) at 3 weeks of age.

### Micro-CT analysis

Micro-computed tomography imaging (µCT) was performed to quantify trabecular architecture, cortical bone geometry, TMD and tibia length. Left tibiae were dissected from all mice, stored in H_2_O at −20°C and thawed prior to scanning with a SkyScan 1272 X-ray microtomograph (Bruker Corporation, Kontich, Belgium). The bones were wrapped in paper tissue and enclosed at a consistent vertical height in tightly fitting rigid plastic tubes filled with water, to prevent movement artefacts. Two images were averaged at each rotation angle to reduce noise. For trabecular bone, images were obtained at a 4.5 µm resolution, using a 0.5 mm aluminium filter and 0.3° rotation step. For cortical bone image accrual and tibial length measurements, images were obtained at a 9 µm resolution using a 0.5 mm aluminium filter with a 0.5° rotation step. Scans were reconstructed using NRecon software (Bruker) and a volume of interest was selected using Data Viewer software (Bruker). Two hundred slices of the metaphysis were taken for analysis of trabecular bone, with the ROI starting 10 slices below the base of the growth plate (GP). One hundred slices of the diaphysis were taken for analysis of cortical bone, with the ROI starting 50 slices above the tibia-fibula junction. CTAn software (Bruker) was used to analyse appropriate parameters. Cortical TMD was calculated using hydroxyapatite rod pair phantoms of a known density (0.25 g/cm^3^ and 0.75 g/cm^3^), which were scanned using the same settings used for cortical image accrual. Tibial length was measured from the proximal tibial plateau to the distal tip of the medial malleolus.

### Bone marrow adiposity

Marrow adiposity was assessed by osmium tetroxide staining of intact right tibiae of 7-week-old mice, as previously described (Scheller et al., 2014). Tibiae were fixed in neutral-buffered formalin for 1 week, scanned by µCT imaging, decalcified in 14% ethylenediaminetetraacetic acid (EDTA) for 2 weeks at 4°C and then stained with 1% osmium tetroxide solution for 48 h before further µCT imaging. The volumes of total, regulated (r) and constitutive (c) BMAT (Ad.V) were determined and calculated as the percentage of the marrow cavity volume (Ma.V), which was determined by µCT prior to staining (Scheller et al., 2015).

### Biomechanical testing

Three-point bending analysis of the cortical region of the left tibiae was carried out immediately after µCT, using a Lloyd LRX5 materials testing machine (Lloyd Instruments, West Sussex, UK). A 100 N loading cell was used with the span fixed at 10 mm and the cross-head lowered at 1 mm/min to determine load to failure and maximum stiffness and deflection of tibiae (Huesa et al., 2011).

### Bone histomorphometry

Two days prior to sacrifice, the mice received an intraperitoneal injection of 30 mg/kg body weight calcein. After dissection, the right femur was fixed in 4% PFA for 24 h, transferred to 70% ethanol and then embedded in methyl methacrylate to allow quantification of longitudinal growth rate at the chondro-osseous junction (Owen et al., 2009; Dobie et al., 2015). Right tibiae were de-calcified for 3 weeks in 10% EDTA and processed to wax using standard procedures. Sections were stained with Toluidine Blue to enable calculation of total and zonal widths of proliferating and hypertrophic zones of the GP (Owen et al., 2009). Static histomorphometry was performed on paraffin-embedded, decalcified sections of tibiae from mice culled at 5 weeks of age. Tartrate-resistant acid phosphatase (TRAP) activity and Fast Red staining was used to identify osteoclasts (TRAP+ve) as multinucleated cells lying on the bone surface. Osteoblasts were visualised by their cuboidal appearance and by the presence of alkaline phosphatase (ALP) activity. Images from 5-week-old mice were scanned using a Nanozoomer slide scanner (Hamamatsu Photonics, Japan) and then the trabecular area of the proximal tibia were analysed. The ROI included only metaphyseal trabecular bone and extended from 50 µm below the GP and within the endocortical bone boundary. Osteoblast and osteoclast numbers per bone surface were determined using BioqantOsteo v 11.2.6 (Bioquant Image Analysis Corp, Nashville, TN, USA), in accordance with the American Society of Bone and Mineral Research Guidelines for nomenclature (Dempster et al., 2013).

### Assessment of chondrocyte proliferation and apoptosis

The ApopTag Plus Fluorescein *In Situ* Apoptosis Detection Kit (EMD Millipore, Bedford, MA, USA) was used as per manufacturer's instructions to quantify the number of apoptotic cells in the tibial GP of 7-week-old mice. Proliferating cell nuclear antigen (PCNA) immunohistochemistry (IHC) was performed using a 1:4000 dilution of an anti-PCNA antibody (Abcam, Cambridge, UK) followed by the Vectastain elite ABC rabbit kit (Vector Laboratories, Peterborough, UK) on paraffin-embedded sections from 3-week-old mice (when rates of proliferation were likely to be highest). Sections were counterstained by Haematoxylin and viewed using a Zeiss AxioImager brightfield microscope. The Haematoxylin-DAB colour deconvolution plugin was used in Fiji to calculate the percentage of PCNA+ve cells in the proliferating zone of the GP of each sample.

### RNA extraction and PCR array

The epiphyses of the right humerus of 7-week-old mice were removed and the shaft centrifuged to remove the bone marrow. Thereafter, cortical bone shaft was snap frozen in liquid nitrogen. RNA was extracted using a modified version of the Qiagen RNeasy kit (Manchester, UK) and reverse transcribed using standard procedures. The cDNA was analysed on the real-time RT^2^ profiler PCR osteogenesis pathway 84-gene array (Qiagen, Manchester UK), in combination with RT^2^ SYBR Green qPCR Mastermix (Qiagen, Manchester, UK) in a Stratagene Mx3000P PCR cycler (Stratagene, CA, USA). Relative gene expression for each gene was calculated in the muscular dystrophy mouse models compared to the WT control group using the 2-ΔΔCT method (Livak and Schmittgen, 2001). ΔCT for each sample was normalised using the geometric mean of the *Gapdh* housekeeping reference gene (included within the array) as this was found to be the most consistent of the 5 housekeeping genes included in the RT^2^ osteogenesis array (*Gusb*, *Actb*, *B2m* and *Hsp90ab1* also tested) across all samples analysed. CT values of >35 cycles were considered non-specific and discarded.

### Immunohistochemistry

A standard indirect IHC procedure was used to detect IGF-1, BMPR1b and MMP-10 expression (Dobie et al., 2015). Primary antibodies were anti-IGF-1 (Abcam, ab9572, 1:500 dilution), BMPR1b (Abcam, ab175385, 1:100 dilution) and MMP-10 (Abcam, ab199688, 1:100 dilution). Control sections were incubated with an equal amount of rabbit IgG in place of the primary antibody. Sections were counterstained by Haematoxylin, mounted and images captured using a Zeiss AxioImager brightfield microscope. Fixation and antigen retrieval were carried out using the same methodology throughout and all IHC for each antibody was performed on the same day under identical conditions, with control specimens also tested for each genotype. After the images were imported into Fiji, the Haematoxylin-DAB colour deconvolution plugin was used to separate the Haematoxylin and DAB components, and the ‘analyse-measure’ tool used to determine the absorbance in a consistent region of each sample, containing both GP and metaphyseal bone. Optical density (OD) was then calculated using the equation: OD=negative (base10)log of mean intensity of transmitted image/illumination (max intensity of image).

Maximum intensity was taken to be 255 for 8-bit images in Fiji (Ruifrok and Johnston, 2001).

### Metatarsal culture

Embryonic (day 18) metatarsal culture was performed as previously described (Houston et al., 2016). The middle three metatarsals were cultured for 10 days and the total length of the metatarsal was measured at day 0, 3, 5 and 7 days using a Nikon Eclipse TE300 microscope with a digital camera attached and Image Tool software (Image Tool Version 3.00, San Antonio, TX, USA).

### Statistical analysis

Mice were identified only by number at the time of culling, to enable blinding by genotype. For clarity, the *mdx:Cmah*^−/−^ mice are referred to as *mdx:Cmah*, and *mdx:Utrn*^+/−^ mice as *mdx:Utrn*. Statistical comparisons were made between mice of each genotype and age, using STATA v15 and GraphPad Prism v7. After checking for normality of data, 1-way ANOVA was used to assess significance of differences between groups and post-test Bonferroni modifications were made to adjust for multiple comparisons. Data are presented as mean±s.d. A *P*-value of <0.05 was accepted as significant. Linear multivariable regression models were used when appropriate to estimate the relationship between the outcome variable under investigation and other independent variables, after adjustment for any potential confounders. Following model generation, regression diagnostics were performed to check the underlying assumptions of the linear model. Residual normality was checked using a normal probability plot and a residual versus fitted plot drawn to ensure that the variance of errors was constant throughout the range of residuals. µCT data were also standardised for tibial length and body weight at cull, but significant results remained the same and therefore only unadjusted data are shown. Quantitative real-time PCR (qPCR) raw data were analysed using the Qiagen web-based PCR array data analysis tool. In accordance with this, *P*-values were calculated based on a Student's *t*-test of the CT data, and a fold change >1 was considered as upregulation and <−1 as downregulation.

This article is part of a special collection ‘A Guide to Using Neuromuscular Disease Models for Basic and Preclinical Studies’, which was launched in a dedicated issue guest edited by Annemieke Aartsma-Rus, Maaike van Putten and James Dowling. See related articles in this collection at http://dmm.biologists.org/collection/neuromuscular.

## Supplementary Material

Supplementary information
