# Supplementary material for: A comparison of the bone and growth phenotype of mdx, mdx:Cmah−/− and mdx:Utrn+/− murine models with the C57BL/10 wild-type mouse
Source: Dis Model Mech. 2020 Jan 10;13(2):dmm040659. doi: 10.1242/dmm.040659 (PMC6994935; doi:10.1242/dmm.040659)
Supplement: Supplementary information [file dmm-13-040659-s1.pdf]

Table S1. Cortical bone parameters of tibiae from WT and muscular dystrophy mouse models as assessed by µCT. Data are presented as mean ± SD (n>6); * denotes p<0.05 compared to WT mice.

Mouse type	3 weeks at cull	5 weeks at cull	7 weeks at cull
**Cortical tissue area, mm² (SD)**			
WT	0.62 (0.09)	0.71 (0.08)	0.94 (0.06)
mdx	0.56 (0.05)	0.81 (0.07)	0.92 (0.10)
mdx:utr$^{+/-}$	0.54 (0.06)	0.81 (0.05)	0.97 (0.08)
mdx:*cmah*$^{-/-}$	0.54 (0.07)	0.76 (0.06)	0.96 (0.08)
**Cortical bone area, mm² (SD)**			
WT	0.29 (0.07)	0.41 (0.07)	0.58 (0.07)
mdx	0.25 (0.03)	0.48 (0.05)	0.60 (0.07)
mdx:utr$^{+/-}$	0.25 (0.03)	0.49 (0.02)	0.63 (0.05)
mdx:*cmah*$^{-/-}$	0.22 (0.03) *	0.46 (0.05)	0.62 (0.07)
**Periosteal perimeter, in mm (SD)**			
WT	3.01 (0.22)	3.16 (0.21)	3.74 (0.11)
mdx	2.85 (0.13)	3.42 (0.16)	3.69 (0.22)
mdx:utr$^{+/-}$	2.81 (0.18)	3.45 (0.10)	3.80 (0.16)
mdx:*cmah*$^{-/-}$	2.78 (0.20)	3.38 (0.19)	3.78 (0.17)
**Endosteal perimeter, in mm (SD)**			
WT	2.20 (0.11)	2.09 (0.09)	2.33 (0.09)
mdx	2.19 (0.11)	2.21 (0.09)	2.21 (0.17)
mdx:utr$^{+/-}$	2.08 (0.09)	2.19 (0.12)	2.23 (0.12)
mdx:*cmah*$^{-/-}$	2.15 (0.19)	2.09 (0.09)	2.22 (0.07)
**Cortical thickness, in mm (SD)**			
WT	0.50 (0.08)	0.52 (0.06)	0.51 (0.08)
mdx	0.46 (0.08)	0.55 (0.02)	0.49 (0.07)
mdx:utr$^{+/-}$	0.48 (0.07)	0.55 (0.03)	0.43 (0.06)
mdx:*cmah*$^{-/-}$	0.54 (0.04)	0.54 (0.02)	0.55 (0.02)
**Mean polar moment of inertia, in mm⁴ (SD)**			
WT	0.05 (0.02)	0.06 (0.02)	0.12 (0.02)
mdx	0.04 (0.01)	0.09 (0.01)	0.12 (0.03)
mdx:utr$^{+/-}$	0.03 (0.01)	0.09 (0.01)	0.14 (0.02)
mdx:*cmah*$^{-/-}$	0.03 (0.01)	0.08 (0.01)	0.13 (0.03)
**Mean eccentricity (SD)**			
WT	0.57 (0.10)	0.55 (0.15)	0.52 (0.09)
mdx	0.47 (0.11)	0.60 (0.07)	0.59 (0.11)
mdx:utr$^{+/-}$	0.61 (0.10)	0.63 (0.03)	0.56 (0.07)
mdx:*cmah*$^{-/-}$	0.58 (0.10)	0.53 (0.06)	0.57 (0.11)

Disease Models & Mechanisms • Supplementary information

Table S2. Trabecular bone parameters of tibiae from WT and muscular dystrophy mouse models as assessed by µCT. Data are presented as mean ± SD (n>6); * denotes $p<0.05$ compared to WT mice.

**Mouse type**	**3 weeks at cull**	**5 weeks at cull**	**7 weeks at cull**
	**Trabecular thickness, in mm (SD)**		
WT	0.03 (0.003)	0.04 (0.003)	0.04 (0.001)
mdx	0.03 (0.002)	0.04 (0.001)	0.04 (0.002)
mdx:utr$^{+/-}$	0.03 (0.002)	0.04 (0.002)	0.04 (0.001)
mdx: *cmah*$^{-/-}$	0.03 (0.001)	0.04 (0.002)	0.05 (0.003) *
	**Trabecular separation, in mm (SD)**		
WT	0.30 (0.05)	0.24 (0.05)	0.18 (0.02)
mdx	0.37 (0.07)	0.26 (0.04)	0.21 (0.02)
mdx:utr$^{+/-}$	0.36 (0.03)	0.30 (0.06)	0.20 (0.02)
mdx: *cmah*$^{-/-}$	0.29 (0.46)	0.23 (0.02)	0.19 (0.01)
	**Trabecular number, in 1/mm (SD)**		
WT	2.04 (0.15)	2.64 (0.43)	3.10 (0.43)
mdx	2.08 (0.42)	2.17 (0.39)	2.67 (0.44)
mdx:utr$^{+/-}$	2.06 (0.38)	1.96 (0.29) *	2.62 (0.45)
mdx: *cmah*$^{-/-}$	2.66 (0.45) *	2.54 (0.35)	2.96 (0.41)
	**Structural Model Index (SD)**		
WT	2.32 (0.08)	2.19 (0.10)	2.14 (0.10)
mdx	2.20 (0.10)	2.31 (0.11)	2.18 (0.07)
mdx:utr$^{+/-}$	2.15 (0.10) *	2.31 (0.06)	2.21 (0.14)
mdx: *cmah*$^{-/-}$	2.19 (0.09)	2.15 (0.13)	2.07 (0.19)
	**Connectivity (SD)**		
WT	618.9 (121.4)	867.3 (221.4)	1042.6 (195.2)
mdx	516.5 (147.2)	702.6 (136.8)	818.1 (207.2)
mdx:utr$^{+/-}$	429.6 (108.8)	554.4 (78.1) *	897.5 (177.2)
mdx: *cmah*$^{-/-}$	561.8 (72.5)	527.5 (107.8) **	723.3 (85.0)***

Table S3. Tibial length from WT and muscular dystrophy mouse models as assessed by µCT. Data are presented as mean ± SD (n>6).

**Mouse type**	**Tibiae length, in mm (SD)**		
	**3 weeks at cull**	**5 weeks at cull**	**7 weeks at cull**
WT	13.02 (0.96)	14.93 (0.18)	16.07 (0.17)
mdx	12.07(0.49)	14.82 (0.59)	16.10 (0.35)
mdx:utr$^{+/-}$	12.42 (0.12)	14.84 (0.84)	16.27(0.30)
mdx:cmah$^{-/-}$	12.45 (0.39)	14.99 (0.53)	16.60 (0.42)

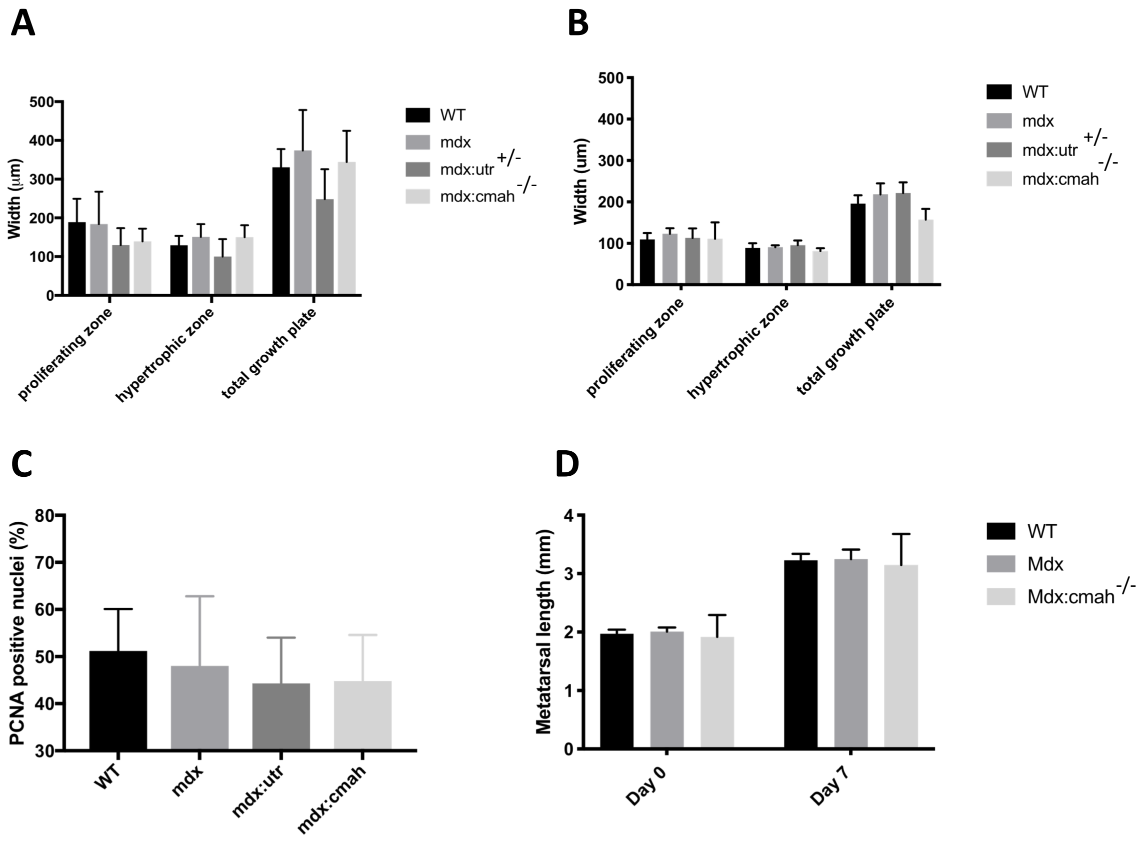

Figure S1. The width of the total growth plate and the individual proliferating and hypertrophic zones were similar at **A)** 3 weeks of age, and **B)** 7 weeks of age. **C)** The percentage of proliferating growth plate chondrocytes was similar in 3-week-old muscular dystrophy and WT mice. Data is presented as mean ± SD (n > 6). **D)** The growth of cultured embryonic (E18) metatarsals from muscular dystrophy and WT mice were similar. Data is presented as mean ± SD (n = 12 metatarsals).
